# Expression of N^6^-methyladenosine (m^6^A) regulators correlates with immune microenvironment characteristics and predicts prognosis in diffuse large cell lymphoma (DLBCL)

**DOI:** 10.1080/21655979.2021.1972644

**Published:** 2021-09-04

**Authors:** Zucheng Xie, Meiwei Li, Haoyuan Hong, Qingyuan Xu, Zhendong He, Zhigang Peng

**Affiliations:** Department of Medical Oncology, First Affiliated Hospital of Guangxi Medical University, Nanning, Guangxi Zhuang Autonomous Region, P. R. China

**Keywords:** m^6^A regulator, immune microenvironment, prognostic signature, DLBCL

## Abstract

This study conducted a comprehensive analysis of the clinical significance of N^6^-methyladenosine (m^6^A) regulators and their relationship with immune microenvironment characteristics in diffuse large cell lymphoma (DLBCL). Consensus clustering was performed to molecularly discriminate DLBCL subtypesbased on m^6^A regulators’ expression. Using the Cox and Lasso regression algorithm, survival-associated m^6^A regulators were identified, and a m^6^A-based prognostic signature was established. The influence of m^6^A risk on immune cell infiltration, immune checkpoint genes, cancer immunity cycle, and immunotherapeutic response was evaluated. Potential molecular pathways related to m^6^A risk were investigated using gene set enrichment analysis. The m^6^A regulators showed satisfactory performance in distinguishing DLBCL subgroups with distinct clinical traits and outcomes. A six m^6^A regulator-based prognostic signature was established and validated as an independent predictor, which separated patients into low- and high-risk groups. High-risk m^6^A indicated worse survival. The B cells naïve, T cells gamma delta, and NK cells resting were the three most affected immune cells by m^6^A risk. Up-regulated (PDCD1 and KIR3DL1) and down-regulated (TIGIT, IDO1, and BTLA) immune checkpoint genes in the high-risk group were identified. The m^6^A risk was found to influence several steps in the cancer immunity cycle. Patients with high-risk m^6^A were more likely to benefit from immunotherapy. Biological function enrichment analysis revealed that high-risk m^6^A to be tended related to malignant tumor characteristics, while low-risk m^6^A showed trend to be related to defensive response processes. Collectively, the m^6^A-based prognostic signature could be a practical prognostic predictor for DLBCL and immune microenvironment characteristics affected by m^6^A may be part of the mechanism.

## Introduction

Latest cancer statistics reported that there were an estimated 81,560 new cases and an estimated 20,720 deaths of non-Hodgkin lymphomas (NHL) in the United State, 2021. NHL ranked 7^th^ and 6^th^ in the morbidity of males and females, respectively, while the mortality ranked 9^th^ for both males and females [1]. Diffuse large cell lymphoma (DLBCL), accounting for 25–30% of all NHL, is a greatly heterogeneous B cell lymphoid neoplasm with substantial variations in genome and genetic alterations, which causes diverse clinical phenotypes and different responses to therapy. Over the past two decades, the combination of rituximab, cyclophosphamide, doxorubicin, vincristine, and prednisone (R-CHOP) has been established as a standard first-line therapy for DLBCL patients based on the results of several phase III clinical trials [2–4]. About 50–70% of DLBCL patients can be clinically cured using the R-CHOP regimen, while the remaining patients end up being either refractory or relapsed. Worse still, only about 10% of the refractory or relapsed DLBCL patients have the fortune to be cured using intensive salvage immunochemotherapy followed by autologous stem cell transplantation, whereas the 90% rest of the patients suffer from dismal outcomes [5,6]. The urgent need for effective therapeutic strategies is unmet and requires unremitting efforts.

With the advent of high-throughput genome sequencing technique, the construction of the genetic landscape of DLBCL has become a reality. It is realizable to decipher which patient is likely or unlikely to benefit from the therapy on genomic level. For example, according to the gene expression profile, DLBCL is mainly divided into two molecularly distinct subtypes: germinal center cell (GCB)-like and activated B cell (ABC)-like DLBCL, which is a milestone for interpreting why DLBCL patients have different responses to the same R-CHOP therapy [7]. Moreover, increasing research has progressively unveiled the pivotal driver genes and pathways in DLBCL, such as TP53, MYC, BCL6, BCL2, MYD88, BCR pathway, NF-κB pathway, PI3K-AKT-mTOR pathway, and JAK-STAT pathway, etc., which help better understand the biological and pathological processes in DLBCL [8]. What’s even more inspiring is growing novel targeted agents such as BTK inhibitor Ibrutinib [9,10], BCL2 inhibitor Venetoclax [11,12], PI3K inhibitor CUDC-907 [13], and AKT inhibitor MK-2206 [14], etc. have shown promising therapeutic potential in relapsed/refractory DLBCL. Therefore, providing a deeper insight into the pathophysiology mechanism in DLBCL is no doubt the essential foundation for the development of novel targeted therapy, which apparently is far from being satisfied yet. As a consequence, research on exploring the DLBCL field from molecular mechanism to clinical application is urgently needed.

Recently, the research on epitranscriptome in cancers has progressed in leaps and bounds owing to the rapid development of high-throughput sequencing such as chromatin immunoprecipitation sequencing, methylated RNA immunoprecipitation sequencing, and assay for transposase-accessible chromatin using sequencing. N^6^-methyladenosine (m^6^A) is one of the common and abundant post-transcriptional modifications in mRNAs, which exerts a pivotal regulatory role in tumorigenesis and progression. The process of m^6^A is mainly achieved by three types of m^6^A regulators: methyltransferases (‘writers’), demethylases (‘erasers’), and binding proteins (‘readers’). m^6^A modifications are assembled by ‘writers’, removed by ‘erasers’, and deciphered by ‘readers’. Increasing studies have revealed the important roles of m^6^A regulators in cancers. For example, methyltransferase METTL3 has been reported as an oncogene involving in the cell proliferation, differentiation, invasion, migration, and apoptosis of various tumors including acute myeloid leukemia, breast cancer, liver cancer, gastric cancer, bladder cancer, prostate cancer, lung cancer, and pancreatic cancer [15]. Demethylase ALKBH5 has been uncovered to exert effects on tumor proliferation, invasion, and metastasis in lung cancer, gastric cancer, pancreatic cancer, colon cancer, glioblastoma, osteosarcoma, and ovarian cancer as an oncogene or tumor suppressor [16]. The ‘readers’ YTH domain-containing proteins, including YTHDF1-3 and YTHDC1-2, have also been proved to be closely in connection with poor prognosis of colorectal cancer, hepatocellular carcinoma, breast cancer, and ovarian cancer [17]. The combination of m^6^A regulators for constructing prognostic model has recently been a novel signature for predicting the outcome of tumor patients. For instance, high-risk m^6^A has been clarified as an unfavorable indicator in clear cell renal cell carcinoma [18], pancreatic cancer [19], non-small cell lung cancer [20], head and neck squamous cell carcinoma [21] gastric cancer [22], hepatocellular carcinoma [23], and colorectal carcinoma [24], which indicated worse survival of patients. However, in DLBCL, no study has reported the combined prognostic value of m^6^A regulators. On the other hand, due to the great heterogeneity of DLBCL, how to accurately discriminate the risk-stratification of a patient is pivotal to decision-making of therapeutic strategies. The current scoring system mainly includes the international prognostic index (IPI), revised-IPI (R-IPI), and National Comprehensive Cancer Network IPI (NCCN-IPI). However, none of the risk score systems could discriminate a patient subgroup with long-term survival clearly <50%. Scholars suggested that we should integrate molecular traits of DLBCL to better characterize high-risk group for which novel therapies are most needed [25]. As a result, it is imperative to investigate the prognostic value of combined m^6^A regulators in DLBCL, which is promising for the development of novel targeted therapy and clinical management of DLBCL patients.

**Figure 1.**
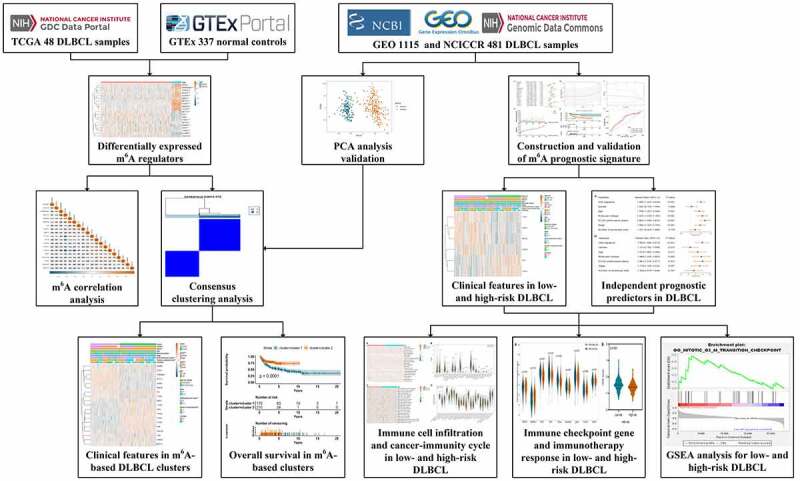
The flowchart of the whole study design

In the current study, we will be the first to comprehensively investigate the prognostic value of m^6^A regulators and construct an m^6^A-based prognostic signature for DLBCL. More importantly, we will provide new perspectives on the relationship between m^6^A risk and tumor immune microenvironment characteristics in DLBCL (Figure 1). We hope the work achieved here will throw light on the interactions between epitranscriptome and immune microenvironment, as well as their clinical potential in DLBCL.

## Methods

### Collection of m^6^A regulators and DLBCL data

Based on currently published literatures [26–29], a total of 22 common m^6^A regulators, which included 8 ‘Writers’ (METTL3, METTL14, METTL5, WTAP, ZC3H13, RBM15, RBM15B, KIAA1429), 2 ‘Erasers’ (ALKBH5, FTO), and 12 ‘Readers’ (YTHDC1, YTHDC2, YTHDF1, YTHDF2, YTHDF3, IGF2BP1, IGF2BP2, IGF2BP3, HNRNPA2B1, HNRNPC, EIF3A, FMR1) were collected. The 22 m^6^A regulators were used for the subsequent analysis.

The Cancer Genome Atlas (TCGA), Genotype-Tissue Expression (GTEx), and Gene Expression Omnibus (GEO) databases were used for obtaining the transcriptome expression and clinical information of DLBCL samples and normal controls. The RNA-seq data of 48 DLBCL samples from the TCGA and 337 normal controls from the GTEx were downloaded, removed batch effect, merged, and normalized with log_2_(FPKM+1). Four mRNA microarrays (GSE10846, GSE31312, GSE87371, and GSE23501) were included in the current study. The expression of mRNAs was normalized with log2 transformation for subsequent analysis. The clinical parameters such as age, gender, stage, extranodal site, ECOG performance status, molecular subtype, LDH ratio, overall survival and survival status were extracted. Patients with an overall survival under 90 days were removed for a better quality of the analysis. Dataset GSE10846 was used as a training set, while the combined data of GSE31312, GSE87371, and GSE23501 was used as an external validation set. A SVA package in R version 4.02 was used for removing batch effects when combining the datasets [30]. Another independent RNA-seq dataset containing 481 samples was downloaded from the National Cancer Institute (NCI) Center for Cancer Research (CCR) of the TCGA program for the use of predicting the potential immunotherapeutic response. The details of the included datasets can be found in Table 1.

**Table 1.** The information of the included datasets in the study

Source	Included DLBCL sample	Included normal controls	Purpose
TCGA	48	0	Investigation of the expression of m^6^A regulators as DLBCL samples
GTEx	0	337	Investigation of the expression of m^6^A regulators as normal controls
GSE10846	380	0	Investigation of DLBCL subtypes based on m^6^A regulators and their clinical significance Construction of m^6^A prognostic signature and investigation of the clinical significance of m^6^A signature Investigation of the influence of m^6^A risk on immune cell infiltration, the activity of cancer immunity cycle, and immune checkpoint genes
GSE31312	454	0	Validation of m^6^A prognostic signature as an external dataset
GSE87371	214	0	Validation of m^6^A prognostic signature as an external dataset
GSE23501	67	0	Validation of m^6^A prognostic signature as an external dataset
NCICCR	481	0	Investigation of immunotherapeutic response in low- and high-risk DLBCL

### Differential expression and correlation analysis for m^6^A regulators

To identify differentially expressed m^6^A regulators in DLBCL, the Wilcoxon signed-rank test was adopted and achieved in R version 4.02. Extremely low-expressed regulators (average expression<0.5) were removed for better data quality. An absolute value of log_2_FC over 1 and a p-value below 0.05 were utilized for screening statistically differentially expressed m^6^A regulators in DLBCL. A violin plot and a heatmap plot achieved with the vioplot and pheatmap packages in R version 4.02 were used to visualize the differentially expressed m^6^A regulators.

In order to understand the correlations among the m^6^A regulators in DLBCL, a Spearman correlation analysis was conducted for the differentially expressed m^6^A regulators using the dataset GSE10846. A corrplot R package was utilized for correlation analysis and visualization. A p-value under 0.05 was regarded as statistically significant.

### Exploration of m^6^A-based DLBCL clusters and their clinical significance

We wondered whether the differentially expressed m^6^A regulators contribute to distinguishing different molecular subtypes of DLBCL. Hence, an unsupervised class discovery approach named Consensus Clustering was applied via a ConsensusClusterPlus R package [31]. The uncovered DLBCL clusters via m^6^A regulators were validated using Principal Component Analysis (PCA) [32]. The correlation between the m^6^A-based clusters and the clinical parameters of DLBCL patients were investigated. The Chi-squared test was applied when estimating the relationship between different m^6^A-based clusters and clinical characteristics, such as age, gender, stage, extranodal site, ECOG performance status, LDH ratio, and molecular subtype. Meanwhile, the Kaplan–Meier survival analysis was utilized to compare the survival of DLBCL patients in different clusters. A statistical p value <0.05 was used to evaluate the difference.

### Construction and validation of m^6^A prognostic signature in DLBCL

The 380 DLBCL samples in GSE10846 were randomly divided into two groups (training set and testing set) equally. In the training set, 190 DLBCL patients were included for the univariate Cox regression analysis to select prognosis-related m^6^A regulators. A p value<0.05 was used as a cutoff to screen statistically significant prognostic m^6^A regulators for further analysis. The Least Absolute Shrinkage and Selection Operator (Lasso) regression algorithm, a popular method for regression analysis with high‐dimensional features [33], was further adopted for variable determination and regularization so as to raise the prediction accuracy and interpretability of the prognostic model. The m^6^A risk stratification was based on the m^6^A risk score, which was computed as follows: risk score = $\sum_{i}^{n}X_{i}*Coef_{i}$. In the formula, the *X_i_* represented the expression of each m^6^A regulator in the model, while *Coef_i_* represented the coefficient of each m^6^A regulator in the Lasso regression analysis. In the testing set, the whole combined samples set of GSE10846, and the external validation set (GSE31312, GSE87371, and GSE23501), the same formula was utilized to calculate the risk score and construct validation models. The DLBCL patients were classified as low-risk and high-risk groups according to a median value of the m^6^A risk score. A survminer package and a survivalROC package in R were used to depict the K-M survival curve and Receiver Operating Characteristic curve (ROC) curve, respectively. Since most of the DLBCL adverse events occur in the first 2 years after diagnosis [34], the area under the curve (AUC) of the time-dependent ROC, which was calculated for evaluating the predictive capacity of the m^6^A signature, was assessed at the time point of 1-, 2-, and 5-year. Moreover, the statistical difference of DLBCL clinical traits (age, gender, stage, extranodal site, ECOG performance status, LDH ratio, and molecular subtype) in low- and high-risk DLBCL was evaluated using Chi-squared test.

To determine whether m^6^A prognostic signature is an independent prognostic factor in DLBCL, univariate and multivariate Cox regression analyses were performed for m^6^A signature along with age, gender, stage, extranodal site, ECOG performance status, LDH ratio, and molecular subtype in GSE10846. Hazard ratio (HR), 95% confidence interval (CI), and p-value were calculated.

### Estimation of tumor immune microenvironment characteristics and immunotherapeutic response using m^6^A signature

We wondered whether m^6^A risk stratification is correlated with tumor immune microenvironment characteristics. As a result, we adopted CIBERSORT, a powerful analytical tool to estimate the abundances of immune cells using gene expression data [35], to identify 22 different kinds of immune cells in the DLBCL samples from GSE10846. Then, we compared the difference of immune cell infiltration between low-risk and high-risk groups using Wilcoxon signed-rank test. Moreover, we investigated the influence of m^6^A risk on the activity of cancer-immunity cycle using the Tracking Tumor Immunophenotype (TIP) web tool [36]. Besides, the expression of nine previously reported immune checkpoint genes (CTLA4, LAG3, TIGIT, HAVCR2, PDCD1, IDO1, VISTA, KIR3DL1, BTLA) [37] in low- and high-risk groups were also investigated. More importantly, we predicted the likelihood of the response to cancer immunotherapy in low- and high-risk groups from an external RNA-seq data using tumor immune dysfunction and exclusion (TIDE) algorithm (http://tide.dfci.harvard.edu/) [38]. A p-value under 0.05 was deemed to be statistically significant.

### Gene set enrichment analysis (GSEA)

GSEA is a knowledge-based computational approach for interpreting a prior defined set of genome-wide expression profiles, which helps determine statistically significant concordant differences of biological function between two phenotypes [39]. As a result, for the purpose of illuminating the underlying molecular pathways related to m^6^A risk, GSEA was performed to select the most significant gene ontology terms (GO) in low- and high-risk groups. GSEA version 4.10 software and Molecular Signatures Database v7.2 were applied for the current analysis.

## Results

### Differentially expressed m^6^A regulators in DLBCL

**Figure 2.**
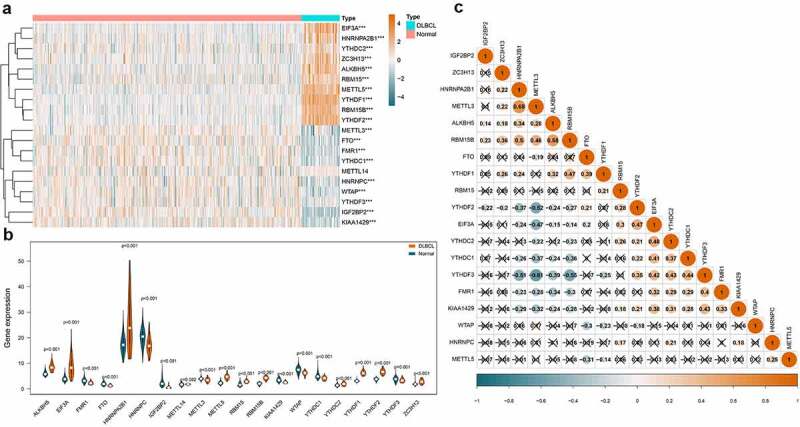
Expression and correlations of m^6^A regulators in DLBCL. A. Expression heatmap of m^6^A regulators in DLBCL and normal controls. Darker blue indicates lower expression, while darker orange indicates higher expression. B. Violin plots of m^6^A regulators in DLBCL and normal controls. C. Correlations among the differentially expressed m^6^A regulators in DLBCL. Darker blue indicates stronger negative correlations, while darker orange indicates stronger positive correlations. The coefficient with a cross glyph on it indicates no statistical significance

Regulator IGF2BP1 (average expression = 0.101) and IGF2BP3 (average expression = 0.441) were removed due to extremely low expression. Finally, the expression of the 20 m^6^A regulators in 48 DLBCL samples and 337 normal controls were compared. As shown in Figure 2(a), the heatmap displayed the expression distribution of the 20 m^6^A regulators. And the violin plot in Figure 2(b) visualized the expression difference of each m^6^A regulator. In summary, 19 out of 20 regulators were differentially expressed, among which, 10 were up-regulated (EIF3A, HNRNPA2B1, YTHDC2, ZC3H13, ALKBH5, RBM15, METTL5, YTHDF1, RBM15B, YTHDF2) and 9 were down-regulated (METTL3, FTO, FMR1, YTHDC1, HNRNPC, WTAP, YTHDF3, IGF2BP2, KIAA1429) in DLBCL. In Figure 2(c), the Spearman correlation analysis was achieved for the 19 differentially expressed m^6^A regulators. We can observe that the correlations among the m^6^A regulators were complicated. For example, the ‘Writers’ can be positively correlated such as METTL3 and RMB15B (coef = 0.46), or negatively correlated such as METTL3 and KIAA1429 (coef = −0.32). An ‘Erases’ (ALKBH5) can be positively associated with a ‘Writers’ (RBM15B) (coef = 0.58). And the ‘Readers’ can also be positively related like YTHDF3 and YTHDC1 (coef = 0.44), or negatively related such as YTHDF3 and HNRNPA2B1 (coef = −0.51). Similarly, a ‘Writer’ can be positively related to a ‘Reader’ such as METTL3 and HNRNPA2B1 (coef = 0.68), or negatively related to another ‘Reader’ such as METTL3 and YTHDF3 (coef = −0.61).

### m^6^A-based DLBCL clusters and clinical implication

**Figure 3.**
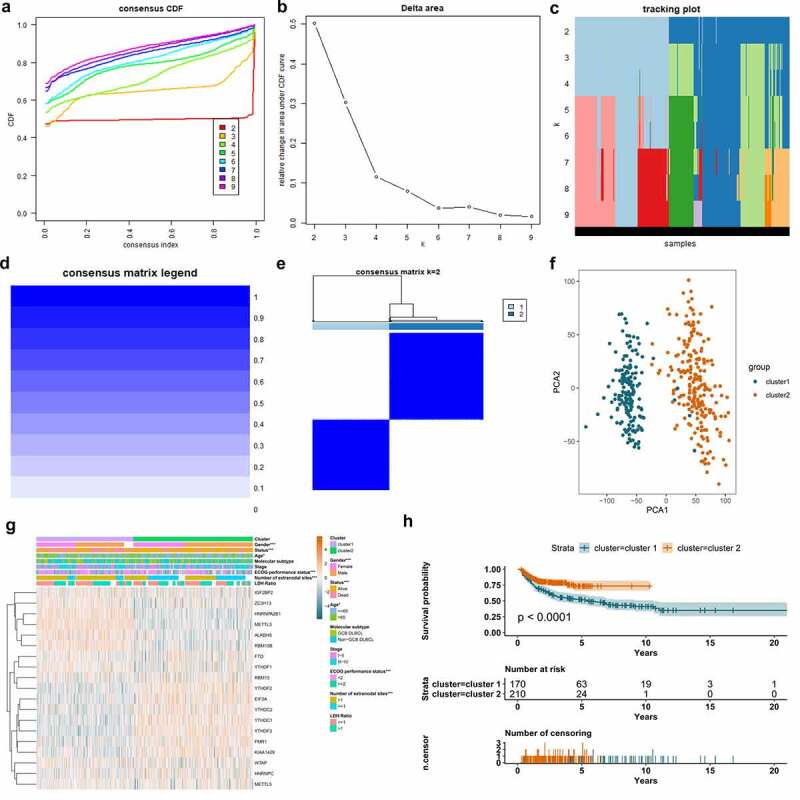
Molecular clusters of DLBCL based on m^6^A regulators and their clinical significance. A. Consensus cumulative distribution function (CDF) plot displays what optimal number of clusters should be determined for yielding the best confidence of consensus and cluster. B. Delta area plot displays the relative change in area under the CDF curve, which helps determine *k* at which there is no appreciable increase. C. Tracking plot shows the cluster assignment of items (horizontal ordinate) for each *k* (vertical coordinate) by colors. D. The graph of consensus matrix color legend. E. Heatmap and dendrogram of the consensus matrix for *k* = 2. The cluster memberships are marked by colored rectangles. F. Principal component analysis (PCA) analysis reveals the reliability of clusters divided by m^6^A regulators. G. The combination of m^6^A expression heatmap and clinical features in m^6^A-based clusters. The clinical parameters gender, age, ECOG performance status, number of extranodal sites, and survival status shows a statistically significant difference in m^6^A-sorted clusters. H. K-M survival curve displays the patients in cluster 2 have better survival than patients in cluster 1

**Figure 4.**
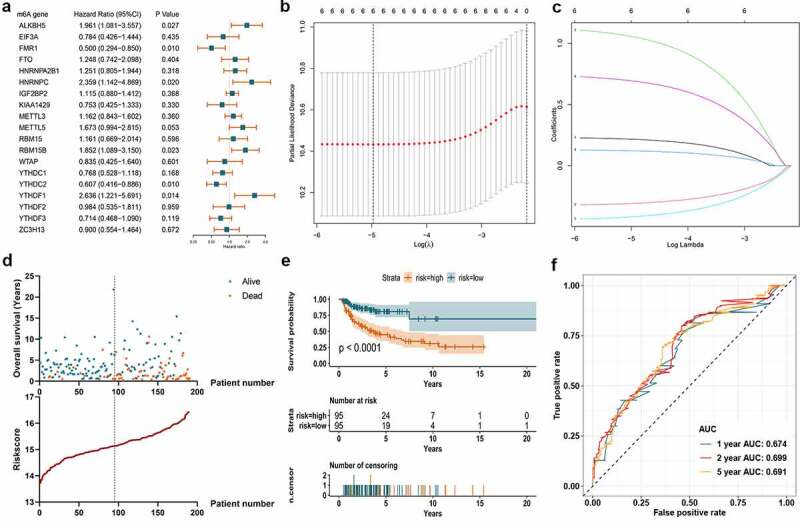
Construction of m^6^A prognostic signature using the Lasso algorithm in the training set. A. Forest plot displays the prognostic value of differentially expressed m^6^A regulators using univariate Cox regression. B. Cross-validation curve for tuning variant selection in the LASSO regularized model. A thousand times across-validations were adopted for determining the best Lambda value. C. Lasso coefficient shrinkage of the six included m^6^A regulators along with larger numbers of log Lambda. Each colored line describes a single predictor and its coefficient score. D. The survival information of the patients in the training set. The upper part displays the survival time and status of each patient, while the lower part displays the m^6^A risk score curve of the patients. E. K-M survival analysis depicts the overall survival of the patients in the training set. The patients in low-risk group have better survivals than those in high-risk group (p < 0.001). F. ROC curve displays the predictive efficacy of the m^6^A signature in the training set. The predictive AUC of 1-, 2-, and 5-year is 0.674, 0.699, 0.691, respectively

**Figure 5.**
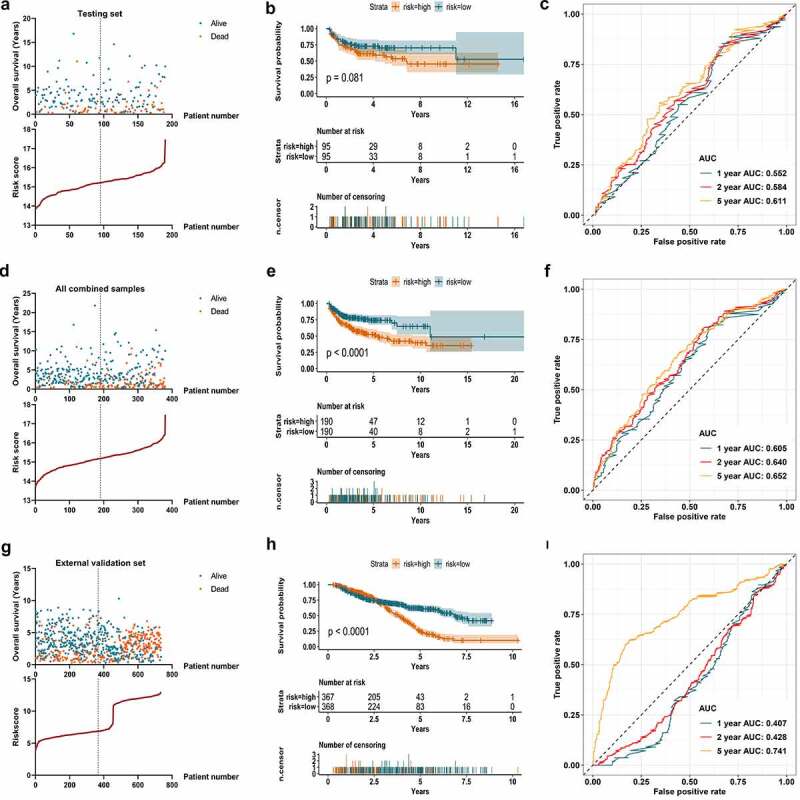
Validation of the m^6^A signature in the testing set, entire combined samples, and external datasets. A. The survival information of the patients in the testing set. The upper part displays the survival time and status of each patient, while the lower part displays the m^6^A risk score curve of the patients. B. K-M survival analysis depicts the overall survival of the patients in the testing set. The patients in low-risk group show a trend to have better survivals than those in high-risk group (p = 0.081). C. ROC curve displays the predictive efficacy of the m^6^A signature in the testing set. The predictive AUC of 1-, 2-, and 5-year is 0.552, 0.584, 0.661, respectively. D. The survival information of all the patients in the combination of training and testing sets. The upper part displays the survival time and status of each patient, while the lower part displays the m^6^A risk score curve of the patients. E. K-M survival analysis depicts the overall survival of all the patients in the combination of training and testing sets. The patients in low-risk group have better survivals than those in high-risk group (p < 0.001). F. ROC curve displays the predictive efficacy of the m^6^A signature in the combination of training and testing sets. The predictive AUC of 1-, 2-, and 5-year is 0.605, 0.640, 0.652, respectively. G. The survival information of the patients in the external validation set. The upper part shows the survival time and status of each patient, while the lower part shows the m^6^A risk score curve of the patients. H. K-M survival analysis displays the overall survival of the patients in the external validation set. The patients in low-risk group have better survivals than those in high-risk group in the follow ups after three years (p < 0.001). I. ROC curve displays the predictive efficacy of the m^6^A signature in the external validation set. The predictive AUC of 5-year is 0.741

As we can observe in Figure 3(a-c) and **Figure S1**, when clustering the samples using a specified cluster count (*k*) from 2 to 9, only *k* = 2 showed the best performance to distinguish the subtypes of DLBCL by the differentially expressed m^6^A regulators. In Figure 3(d-e), the heatmap showed that the samples in cluster 1 were highly correlated, so were those in cluster 2. There was barely any correlation significant between cluster 1 and cluster 2, which indicated that m^6^A regulators were capable to differentiate the DLBCL samples into molecularly distinguishable subtypes. The finding was validated using the PCA analysis, which was displayed in Figure 3(f)Figure 4. The samples in cluster 1 and cluster 2 were both highly clustered, which further proved that m^6^A-based DLBCL clusters are reliable. The clinical significance of the two m^6^A-based DLBCL clusters was also investigated and demonstrated in Figure 5(g-h). Figure 5(g) displayed the expression heatmap of the differentially expressed m^6^A regulators in m^6^A-based clusters and their correlation with clinical parameters in DLBCL. The gender, age, ECOG performance status, number of extranodal site, and survival status showed statistical differences in cluster 1 and cluster 2 (p < 0.05). In Figure 5(h), the survival of DLBCL patients in cluster 1 and cluster 2 also showed statistical difference. The overall survival rate of DLBCL patients in cluster 2 outperformed those in cluster 1 (p < 0.001).

### m^6^A signature and clinical implication

Univariate Cox regression algorithm was carried out for the 19 differentially expressed m^6^A regulators. Six out of the 19 m^6^A regulators (ALKBH5, FMR1, HNRNPC, RBM15B, YTHDC2, and YTHDF1) showed statistically prognostic value in DLBCL patients (Figure 4(a)). Through further Lasso regression, all the six prognostic m^6^A regulators were determined for the construction of prognostic signature (Figure 4(b-c)). The detailed calculation of risk score was: risk score = 0.21642 × Expression of ALKBH5-0.30866 × Expression of FMR1 + 1.05555 × Expression of HNRNPC+0.12185 × Expression of RBM15B-0.41769 × Expression of YTHDC2 + 0.69323 × Expression of YTHDF1. The calculated risk score curve and survival information of the patients in the training set were displayed in Figure 4(d). The K-M survival curve suggested the patients with low-risk m^6^A had higher overall survival rate than those in high-risk group in the training set (Figure 4(e)). ROC curve revealed the AUC at 1-, 2-, and 5-year was 0.674, 0.699, and 0.691, which suggested certain predictive capacity of the m^6^A signature in the training set (Figure 4(f)).

**Figure 6.**
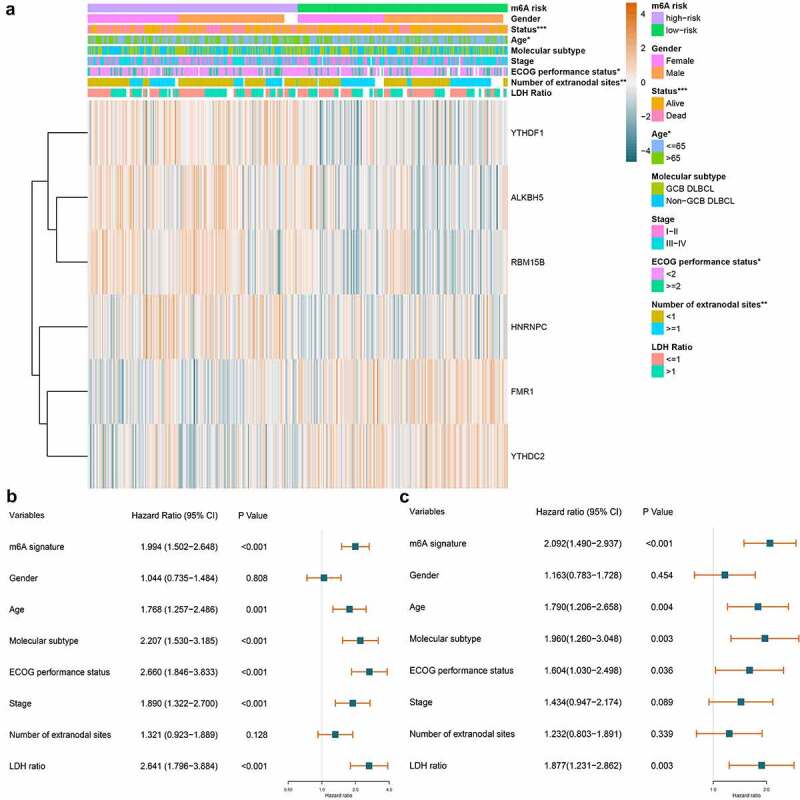
The relationship between m^6^A prognostic signature and clinical parameters of DLBCL. A. The combination of m^6^A expression heatmap and clinical parameters in low- and high-risk groups. The clinical parameters age, ECOG performance status, number of extranodal sites, and survival status shows statistical significance in low- and high-risk groups. B. Forest plot displays the prognostic value of m^6^A signature and other clinical parameters of DLBCL using univariate Cox regression. m^6^A signature (p < 0.001), age (p = 0.001), molecular subtype (p < 0.001), ECOG performance status (p < 0.001), stage (p < 0.001), and LDH ratio (p < 0.001) show statistically significant difference in low- and high-risk groups. C. Forest plot displays the independent predictors in DLBCL using multivariate Cox regression. m^6^A signature (p < 0.001), age (p = 0.004), molecular subtype (p = 0.003), ECOG performance status (p = 0.036), and LDH ratio (p = 0.003) are validated as independent prognostic predictors in DLBCL

The m^6^A prognostic signature in the training set was validated using a testing set and a combined data of the training set and testing set as a whole. In Figure 5(a), the risk score curve and survival information of patients in the testing set were visualized. Although there was no statistical significance when conducting a K-M survival analysis for the samples in the testing set (p = 0.081), it still showed an obvious trend that patients with low-risk m^6^A had better survival (Figure 5(b)). The AUC of the 1-, 2-, and 5-year achieved 0.552, 0.584, and 0.611 in the testing samples (Figure 5(c)). Figure 5(d) displayed the calculated risk score and survival information of the combined training set and testing set. A statistically significant difference was observed in the K-M survival curve analysis for the combined samples (p < 0.001). Patients with low-risk m^6^A showed better survival than those in high-risk group (Figure 5(e)). Figure 5(f) displayed the predictive efficacy of the m^6^A signature, which yielded an AUC for 1-, 2-, and 5-year of 0.605, 0.640, and 0.652, respectively. In the external validation set (735 samples), the survival information and m^6^A risk score of the patients were shown in Figure 5(g). The survival rate did not show distinct difference in low- and high-risk groups before the first three years. However, three years later, the patients with low-risk m^6^A began to show better survival than those with high-risk m^6^A, which indicated the long-term effect of m^6^A risk (Figure 5h). The predictive AUC of 5-year was 0.741 (Figure 5i). Regarding the clinical parameters, age, ECOG performance status, number of extranodal sites, and survival status showed statistical significance in low- and high-risk groups (Figure 6a).

Whether m^6^A signature is an independent factor in DLBCL was investigated. Displaying in Figure 6b, the univariate Cox regression revealed that m^6^A signature (HR = 1.994, p < 0.001), age (HR = 1.768, p = 0.001), molecular subtype (HR = 2.207, p < 0.001), ECOG performance status (HR = 2.660, p < 0.001), stage (HR = 1.890, p < 0.001), and LDH ratio (HR = 2.641, p < 0.001) were all statistically significant prognostic predictors. And multivariate Cox regression analysis displayed in Figure 6c further verified that m^6^A signature (HR = 2.092, p < 0.001), age (HR = 1.790, p = 0.004), molecular subtype (HR = 1.960, p = 0.003), ECOG performance status (HR = 1.604, p = 0.036), and LDH ratio (HR = 1.877, p = 0.003) were all independent prognostic predictors in DLBCL.

### Estimation of m^6^A risk and tumor immune microenvironment characteristics

**Figure 7.**
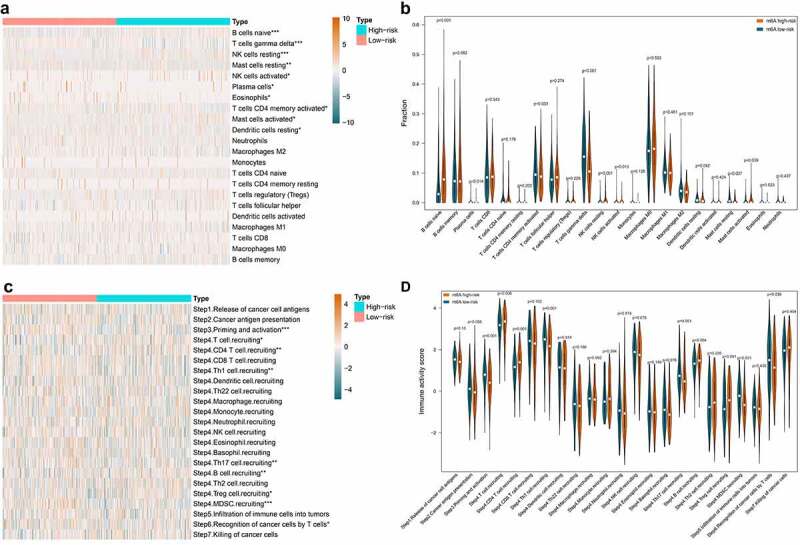
The effect of m^6^A risk on immune cell infiltration and immunity cycle activity. A. Heatmap visualizes immune cell infiltration degree in each DLBCL samples. Statistically significant immune cell infiltration in low-risk vs. high-risk DLBCL was marked with an asterisk (p < 0.001***, p < 0.005**, p < 0.05*). B. Violin plot displays the difference of each type of immune cell in low-risk vs. high-risk DLBCL. C. Heatmap visualizes the activity of each step in cancer immunity cycle in each DLBCL samples. Statistically significant steps in cancer immunity cycle in low-risk vs. high-risk DLBCL was marked with an asterisk (p < 0.001***, p < 0.005**, p < 0.05*). D. Violin plot displays the difference of each step in cancer immunity cycle in low-risk vs. high-risk DLBCL

The immune cell infiltration in low- and high-risk groups was explored. The bar plot in **Figure S2** visualized the proportion of the 22 immune cells in each DLBCL sample on the whole. And Figure 7a also displayed the 22 immune cells’ distribution in low- and high-risk groups as a heatmap. The immune cell infiltration difference in low-risk vs. high-risk groups was compared by statistics. As shown in Figure 7b, ten out of 22 immune cells showed statistical differences between the low- and high-risk groups. The B cell naïve, NK cells resting, NK cells activated, and Mast cells activated were significantly up-regulated in high-risk group. The Plasma cells, T cells CD4 memory activated, T cells gamma delta, Dendritic cells resting, Mast cells resting, and Eosinophils were significantly down-regulated in high-risk group. Integrating some immunological studies [40–42], the immunological mechanism and final effect against tumor of the top three altered lymphocytes were shown in Table 2. We can notice that alternations of immune cell infiltration caused by m^6^A risk did not always associate with anti-tumor effect. The correlations among the 10 differentially distributed immune cells were depicted in **Figure S3**. We can notice that T cells CD4 memory activated was positively correlated with T cells gamma delta (coef = 0.41), while negatively correlated with B cell naïve (coef = −0.35). The T cells gamma delta was negatively related to B cell naïve (coef = −0.44) and NK cells resting (coef = −0.49).

**Table 2.** Immune effects of the top three altered lymphocytes

Immune cells	Alteration in high-risk m^6^A group	Study	Mechanism	Effect to anti-cancer immune
B cell naïve	Up-regulated	Diana Stoycheva et al. [40]	B cells exert anti-tumor effects by secreting antibodies and cytokines, processing and presenting antigens, and modulating T cells and other immune cells	Suppression
T cells gamma delta	Down-regulated	Ghita Chabab et al. [41]	γδ T cells display pro-tumor activities with the help of TGF-β, IL-4, and IL-21	Promotion
NK cells resting	Up-regulated	Jacob A Myers et al. [42]	NK cell play anti-cancer effects via the ‘missing-self ’ mechanism, ADCC effect, and producing pro-inflammatory cytokines such as IFNγ and TNF	Suppression

The effect of m^6^A risk on the activity of cancer-immunity cycle was also investigated. As shown in Figure 7c, the heatmap displayed the distribution of the activity of seven stepwise events of anti-cancer immune response in low- and high-risk groups. The immune activity scores of Step 3 (Priming and activation), Step 4 (Th1 cell recruiting, Th17 cell recruiting, MDSC recruiting), and Step 6 (Recognition of cancer cells by T cells) were all significantly lower in high-risk group compared with low-risk group. The immune activity scores of Step 4 (T cell recruiting, CD4 T cell recruiting, B cell recruiting, Treg cell recruiting) were significantly higher in high-risk group compared with low-risk group (Figure 7d).

**Figure 8.**
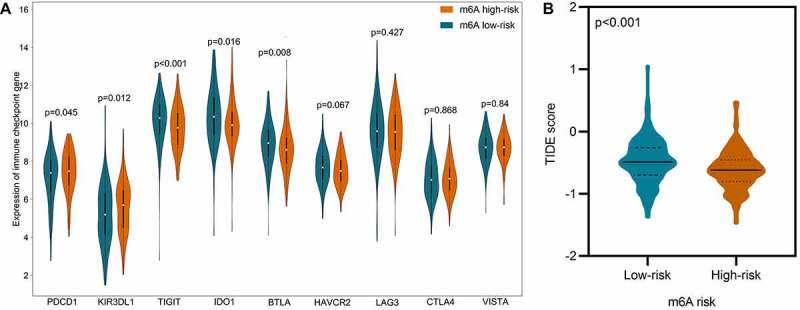
Comparison of immune checkpoint genes and potential immunotherapeutic response in low- and high-risk DLBCL. A. Violin plot displays the expression difference of immune checkpoint genes in low-risk vs. high-risk DLBCL. B. Violin plot displays the difference of TIDE score in low-risk vs. high-risk DLBCL. The TIDE score in high-risk group is significantly decreased, which indicates lower potential of tumor evasion, thus more likely to benefit from immunotherapy

Immune checkpoint is a critical mechanism of tumor immune escape, which makes it a prospective target for cancer immunotherapy. Therefore, the expression of nine well-studied immune checkpoint genes in the low- and high-risk group was analyzed and displayed in Figure 8a. We found that PDCD1 (P = 0.045) and KIR3DL1 (P = 0.012) were significantly increased in high-risk group, while TIGIT (p < 0.001), IDO1 (p = 0.016), and BTLA (p = 0.008) were significantly decreased instead. Regards LAG3, CTLA4, VISTA, and HAVCR2, no statistical significance was observed.

The response to immunotherapy in low- and high-risk groups were predicted using an external independent cohort of 481 samples. As displayed in Figure 8b, the TIDE score in low-risk group surpassed that in high-risk group, which indicates higher potential of tumor evasion, thus less likely to benefit from immunotherapy.

### Enriched pathways in low- and high-risk DLBCL

**Figure 9.**
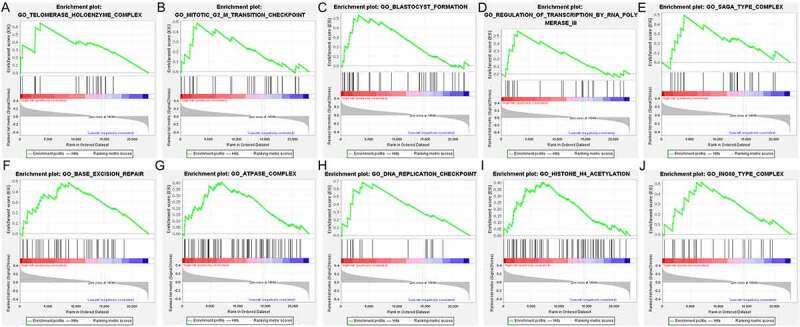
The enriched GO terms in high-risk DLBCL. The top part in each plot displays the enrichment score (ES) of each gene. The middle part of each plot displays the leading edge subset, in which a vertical line represents a single gene. The bottom part shows the distribution of ranking metric scores, in which the red section is positively correlated with high-risk patients, while the blue section is negatively correlated with low-risk patients. A. Enrichment plot of Telomerase holoenzyme complex. B. Enrichment plot of Mitotic G2-M transition checkpoint. C. Enrichment plot of Blastocyst formation. D. Enrichment plot of Regulation of transcription by RNA polymerase III. E. Enrichment plot of Saga type complex. F. Enrichment plot of Base excision repair. G. Enrichment plot of ATPase complex. H. Enrichment plot of DNA replication checkpoint. I. Enrichment plot of Histone H4 acetylation. J. Enrichment plot of INO80 type complex

**Figure 10.**
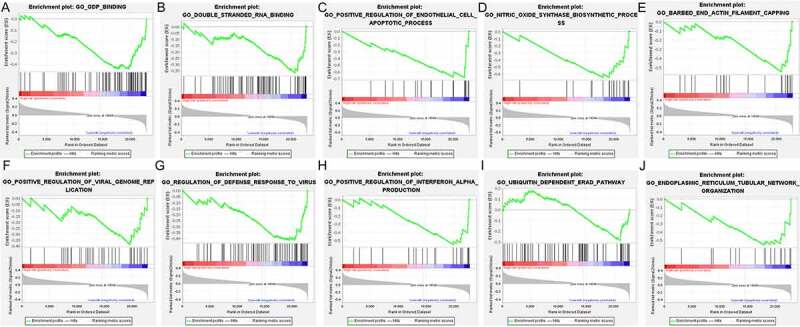
The enriched GO terms in low-risk DLBCL. The top portion in each plot demonstrates the enrichment score (ES) of each gene. The middle portion of each plot displays the leading edge subset, in which a vertical line represents a single gene. The bottom portion shows the ranking metric scores distribution, in which the red section is positively correlated with high-risk patients, while the blue section is negatively correlated with low-risk patients. A. Enrichment plot of GDP binding. B. Enrichment plot of Double stranded RNA binding. C. Enrichment plot of Positive regulation of endothelial cell apoptotic process. D. Enrichment plot of Nitric oxide synthase biosynthetic process. E. Enrichment plot of Barbed end actin filament capping. F. Enrichment plot of Positive regulation of viral genome replication. G. Enrichment plot of Regulation of defense response to virus. H. Enrichment plot of Positive regulation of interferon Alpha production. I. Enrichment plot of Ubiquitin dependent ERAD pathway. J. Enrichment plot of Endoplasmic reticulum tubular network organization

To reveal the difference of potential biological function between the low-risk and high-risk groups, GSEA analysis was carried out. The top 10 significantly enriched terms in the low-risk group and high-risk group were selected for displaying, respectively. As we can see in Figure 9, the top 10 significantly enriched terms in high-risk group included Telomerase holoenzyme complex, Mitotic G2-M transition checkpoint, Blastocyst formation, Regulation of transcription by RNA polymerase III, Saga type complex, Base excision repair, ATPase complex, DNA replication checkpoint, Histone H4 acetylation, INO80 type complex. And GDP binding, Double stranded RNA binding, Positive regulation of endothelial cell apoptotic process, Nitric oxide synthase biosynthetic process, Barbed end actin filament capping, Positive regulation of viral genome replication, Regulation of defense response to virus, Positive regulation of interferon Alpha production, Ubiquitin dependent ERAD pathway, and Endoplasmic reticulum tubular network organization were the top 10 significantly enriched items in low-risk group (Figure 10).

## Discussion

Emerging evidence substantiated that m^6^A modification exerts indispensable regulatory effects in neuronal disorders, osteoporosis, metabolic disease, viral infection, and various cancers [43,44]. Targeting m^6^A regulators for cancer therapy has been a closely focused field by scholars. For instance, Huang et al. recently developed two promising FTO inhibitors named FB23 and FB23-2 using structure-based rational design. Encouragingly, FB23-2 was found to dramatically inhibit proliferation and enhance the differentiation/apoptosis of acute myeloid leukemia (AML) cell line in vitro. More importantly, in vivo experiment, FB23-2 could significantly suppress the progression of AML primary cells in xeno-transplanted mice [45]. In recent years, the prominent role of m^6^A modification in cancer immunotherapy has been gaining more and more attention. Han et al. recently discovered that m^6^A-binding protein YTHDF1 could control anti-tumor immunity by recognizing m^6^A-marked transcripts encoding lysosomal proteases to increase their translation in dendritic cells. Specifically, the deficiency of YTHDF1 elevated the cross-presentation of tumor antigens and antigen-specific CD8 + T cell antitumor response. More remarkably, loss of YTHDF1 enhanced the therapeutic efficacy of PD-L1 checkpoint blockade in vivo [46]. Despite the rapid development of m^6^A research in cancer, few research works have reported the pathological role of m^6^A regulators and their clinical significance in DLBCL. Cheng et al. once reported that down-regulated methyltransferase METTL3 functionally inhibited the DLBCL cell proliferation through reducing the m^6^A methylation and total mRNA level of pigment epithelium-derived factor [47]. Another study revealed that up-regulated piRNA-30473 was associated with aggressive phenotype and poor prognosis of DLBCL patients by virtue of m^6^A dependent regulatory manners. Mechanistically, piRNA-30473 exerted its oncogenic effect via increasing the expression of methylase WTAP and its critical target gene HK2, thus enhanced the global m^6^A level [48]. Nevertheless, the above studies only focused on a single m^6^A regulator, lacking a comprehensive understanding of the potential clinical value of different m^6^A regulators as a whole. Given that, our current study primarily focused on evaluating the prognostic value of 22 known m^6^A regulators, constructing a prognosis predictive model, and investigating the influence on immune microenvironment characteristics of m^6^A risk in DLBCL.

The majority of m^6^A regulators showed differential expression in DLBCL and their interactions were complicated. The same m^6^A regulator could be positively correlated with a regulator while negatively correlated with another regulator. This indicated the m^6^A regulatory network in DLBCL was sophisticated, which deserves further analysis. More interestingly, we found that the DLBCL patients could be divided into two clusters based on differentially expressed m^6^A regulators. More importantly, the gender, age, extranodal site, ECOG performance status, and overall survival of the patients in the two clusters showed statistically significant differences. The patients in cluster 1 had worse survival. Our results were consistent with Shen’s findings, which discovered that m^6^A-based subtypes were significantly associated with overall survival in pan-cancer including DLBCL [49]. As we all known, DLBCL can be mainly divided into two subtypes namely ABC-DLBCL and GCB-DLBCL according to gene expression profile. ABC-DLBCL is associated with more malignant biological properties and worse clinical outcomes than GCB-DLBCL. Population-based studies showed evidence that the 5-year overall survival rate of ABC-DLBCL patients is 35%, while it is 60% for GBC-DLBCL patients [50]. This suggested more positive therapy and follow-up measures should be taken when managing ABC-DLBCL patients. Similarly, different m^6^A-based DLBCL clusters also had distinct overall survival, which is worthy of clinical attention. Since the patients in cluster 1 and ABC-DLBCL subtype both suffered from worse survival, their relationship is worth further investigation. Why ABC-DLBCL is more refractory was deemed to be associated with constitutive activation of the NF-κB and BCR signaling pathways [51]. Whether m^6^A regulators are implicated in the NF-κB and BCR signaling pathways, resulting in corresponding clinical phenotype features also bears thinking about.

Another highlight achieved in the current study was we constructed an m^6^A-based prognostic model for predicting the overall survival of DLBCL patients. Patients with high-risk m^6^A were verified to have poorer outcomes. m^6^A risk scoring model was validated as an independent prognostic predictor, which is as significant as age, molecular subtype, ECOG performance status, and LDH ratio. Scholars have previously constructed prognostic signatures for DLBCL using other predictors. For example, Zhou et al. constructed a prognostic immunoscore model using immune cell infiltration, yielding an AUC of 0.562 in DLBCL [52]. And Hu et al. constructed an integrated prognostic model of a pharmacogenomic gene signature for DLBCL with a predictive AUC of 0.67 [53]. Moreover, Zhang et al. utilized a combined five types of alternative splicing events to construct prognostic predictors for DLBCL patients, which showed an AUC of 0.564 [54]. In the current study, the 5-year predictive AUC of m^6^A signature in the 380 DLBCL patients were 0.652, while it achieved 0.741 in the external validation set of 735 cohorts. Compared with previously published prognostic models, the m^6^A signature showed satisfactory predictive performance, which is qualified for potential clinical application.

The concept of tumor microenvironment (TME) has been come up with for years, and its development has never been kept from moving with the times. It is now clear that TME consists of tumor cells, immune cells, stromal cells, endothelial cells, and cancer-associated fibroblasts [55]. Although infiltrated by immune cells, the cancer cells can somehow escape from immune supervision and destruction through multiple tricky mechanisms. The tumor immune privilege mechanisms mainly include reduced expression of cancer antigens and major histocompatibility complex class I, elevated expression of immune checkpoints, as well as increased recruitment of immunosuppressive cells, such as T regulatory cells, tumor-associated macrophages, and myeloid-derived suppressor cells, etc. [56]. Immune blockades have been developing vigorously over the years, among which, PD-1 is the living proof. PD-1 inhibitor, mainly putting brakes on unrestricted cytotoxic T effector function, was first approved by U.S. Food and Drug Administration for the treatment of unresectable/metastatic melanoma cancer and non-small-cell lung cancer second-line alternative supported by National Comprehensive Cancer Network guideline [57]. However, in relapsed/refractory DLBCL, PD-1 blockade therapy has been disappointing, achieving an objective response of merely 36% [58]. As a result, making immune blockades a success in treating DLBCL is still challenging, while taking a deeper insight into the immune microenvironment characteristics is an essential step.

Plenty of studies have unveiled a close connection between m^6^A and immune microenvironment characteristics. For instance, Shen et al. found that m^6^A modifications contributed to immune regulation in HCC, which were promising to act as novel prognostic predictors and immune therapeutic targets [59]. In gastric cancer, scholars discovered that m^6^A modulation patterns were crucial for TME diversity and complexity. Patients with lower m^6^A score yielded therapeutic advantages and clinical benefits [60]. Xu et al. discovered that m^6^A-related lncRNA is promising biomarkers for predicting immunotherapeutic responses in LUAD [61]. Our current study also provided novel findings on the relationship between m^6^A and immune cell infiltration in DLBCL. We discovered a bunch of up-regulated (B cell naïve, NK cells resting, NK cells activated, and Mast cells activated) and down-regulated (Plasma cells, T cells CD4 memory activated, T cells gamma delta, Dendritic cells resting, Mast cells resting, and Eosinophils) immune cells in high-risk patients. And immune checkpoint-related genes could be up-regulated (PDCD1 and KIR3DL1) or down-regulated (TIGIT, IDO1, and BTLA) in high-risk group, which implied complicated mechanism. Cao et al. have comprehensively reviewed the sophisticated relationship among cancer epigenetics, tumor immunity, and immunotherapy, which suggested tremendous potential of epigenetic therapies [62]. Our current study also investigated the relationship among m^6^A risk, the activity of cancer immunity cycle, and the response to immunotherapy. Our results showed that m^6^A risk did significantly influence the activity of priming and activation of effector T cells, recognition of cancer cells by T cells and the trafficking of some immune cells. High risk of m^6^A signature were discovered to be more likely to response to immunotherapy. This indicated the potential clinical implication of m^6^A signature in prediction of immunotherapeutic response.

Whether the underlying biological function and molecular pathways are different between low-risk and high-risk DLBCL is a noteworthy aspect. Therefore, we explored the possible molecular mechanism in low- and high-risk DLBCL. We discovered that the enriched biological function in high-risk group mainly included cell cycle, DNA replication, transcription, post-transcriptional modification, and DNA repair relative pathways, which were manifestations of malignant tumor features. And in low-risk group, the enriched biological functions included several interesting items such as Positive regulation of endothelial cell apoptotic process, Nitric oxide synthase biosynthetic process, Regulation of defense response to virus, and Positive regulation of interferon Alpha production, which were mostly associated with defensive processes. In detail, we know that anti-angiogenesis is an important perspective for cancer therapy, in which inducing the apoptosis of vascular endothelial cells is a pivotal process [63–65]. And Nitric oxide has been found to be involved in the immune response. Its important synthase NOS2 could regulate macrophages, T cells, B cells, and myeloid-derived suppressor cells [66]. Interferon Alpha has been approved for the treatment of more than 14 types of cancers, including hairy cell leukemia, melanoma, and renal cell carcinoma, as an immune-based oncologic drug for years [67,68]. Collectively, these might help explain why patients in high-risk m^6^A group suffered from worse survival than those in low-risk group on molecular mechanism level. Still, experimental experiments are worthy and necessary to be carried out for further validation.

Despite the highlights of m^6^A-based prognostic signature and their correlation with tumor immune microenvironment characteristics, several limitations should be acknowledged in this study. First, even though we investigated the mRNA expression of the m^6^A regulators, their protein expression should also be verified by immunohistochemistry. Second, although we investigated the influence of m^6^A risk on some immune microenvironment characteristics, such as immune cell infiltration, immune checkpoint-related genes, cancer immunity cycle, and immunotherapeutic response, other important immune characteristics like tumor mutation burden, microsatellite instability, neoantigen, etc. should also be comprehensively analyzed in the future. Besides, the current study design and results were based on bioinformatics analysis. Future experimental studies are required to validate the results and elaborate the exact molecular regulatory mechanism between m^6^A regulators and tumor immune microenvironment characteristics.

## Conclusions

This study comprehensively investigated the clinical significance of multiple m^6^A regulators and established a novel m^6^A risk scoring signature for predicting the survival of DLBCL patients for the first time, which achieved satisfactory predictive performance. More importantly, we unveiled several highlights of immune cell infiltration, immune checkpoint genes, cancer immunity cycle, immunotherapeutic response, and underlying molecular pathways in low- and high-risk DLBCL pioneeringly, which shed light on the potential regulatory relationship between m^6^A and immune microenvironment characteristics. Nevertheless, the obtained results especially the exact mechanism on how m^6^A affects immune microenvironment characteristics still need further experimental research. Anyhow, the findings in this study provided whole new perspectives for the m^6^A epitranscriptome and immunomics in DLBCL, which are promising for the development of individualized and comprehensive management of DLBCL patients.

## Supplementary Material

Supplemental Material

## Data Availability

The clinical information, RNA-seq, and microarray expression data were obtained from the GEO (https://www.ncbi.nlm.nih.gov/geo/), GTEx (https://gtexportal.org/home/), and TCGA (https://portal.gdc.cancer.gov/) databases, which are all publicly available.

## Highlights

1. m^6^A regulators can class the DLBCL into two subgroups with distinct clinical traits and outcomes.
2. m^6^A-based prognostic signature is an independent prognostic predictor in DLBCL.
3. m^6^A-based prognostic signature is related to immune cell infiltration, cancer-immunity cycle, and immune checkpoint genes’ expression.
4. m^6^A-based prognostic signature is potential to predict the response of immunotherapy.
