# Supplementary material for: Expression of N6-methyladenosine (m6A) regulators correlates with immune microenvironment characteristics and predicts prognosis in diffuse large cell lymphoma (DLBCL)
Source: Bioengineered. 2021 Sep 4;12(1):6115–33. doi: 10.1080/21655979.2021.1972644 (PMC8806613; doi:10.1080/21655979.2021.1972644)
Supplement: Supplemental Material [file KBIE_A_1972644_SM0980.zip › supplementary/Supplementary information - figure legends.docx]

**Supplementary information**

**Additional file 1.** Figure S1. Heatmap and dendrogram of the consensus matrix for k from 3 to 9. The cluster memberships were marked by colored rectangles.

**Additional file 2.** Figure S2. Bar plot visualizes the proportion of each immune cell in each DLBCL sample.

**Additional file 3.** Figure S3. Correlations among the 10 differentially distributed immune cells.
