# Supplementary figures and images for: Expression of N6-methyladenosine (m6A) regulators correlates with immune microenvironment characteristics and predicts prognosis in diffuse large cell lymphoma (DLBCL)
Source: Bioengineered. 2021 Sep 4;12(1):6115–33. doi: 10.1080/21655979.2021.1972644 (PMC8806613; doi:10.1080/21655979.2021.1972644)

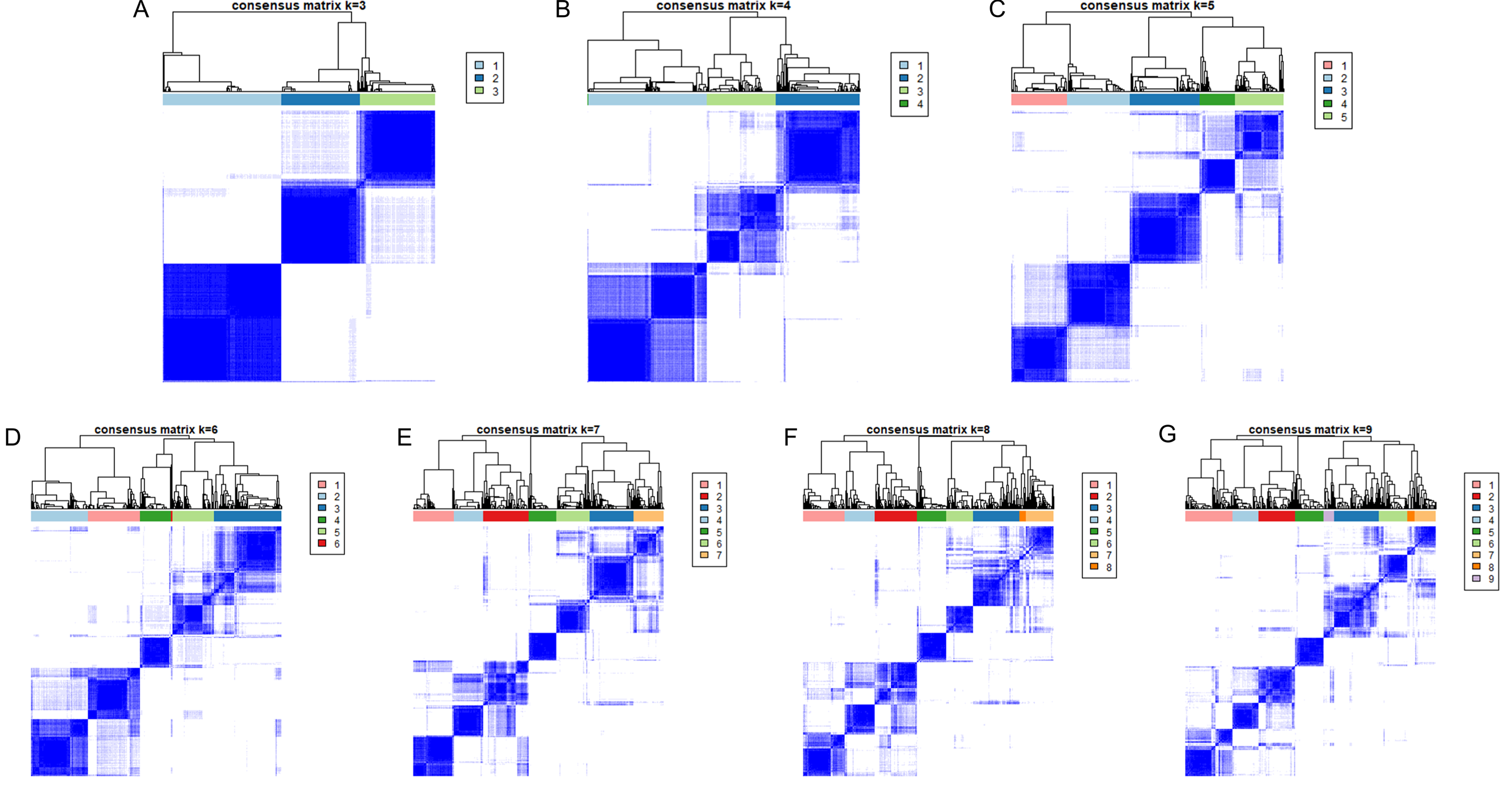

Supplement: Supplemental Material [file KBIE_A_1972644_SM0980.zip › supplementary/Figure S1.jpg]

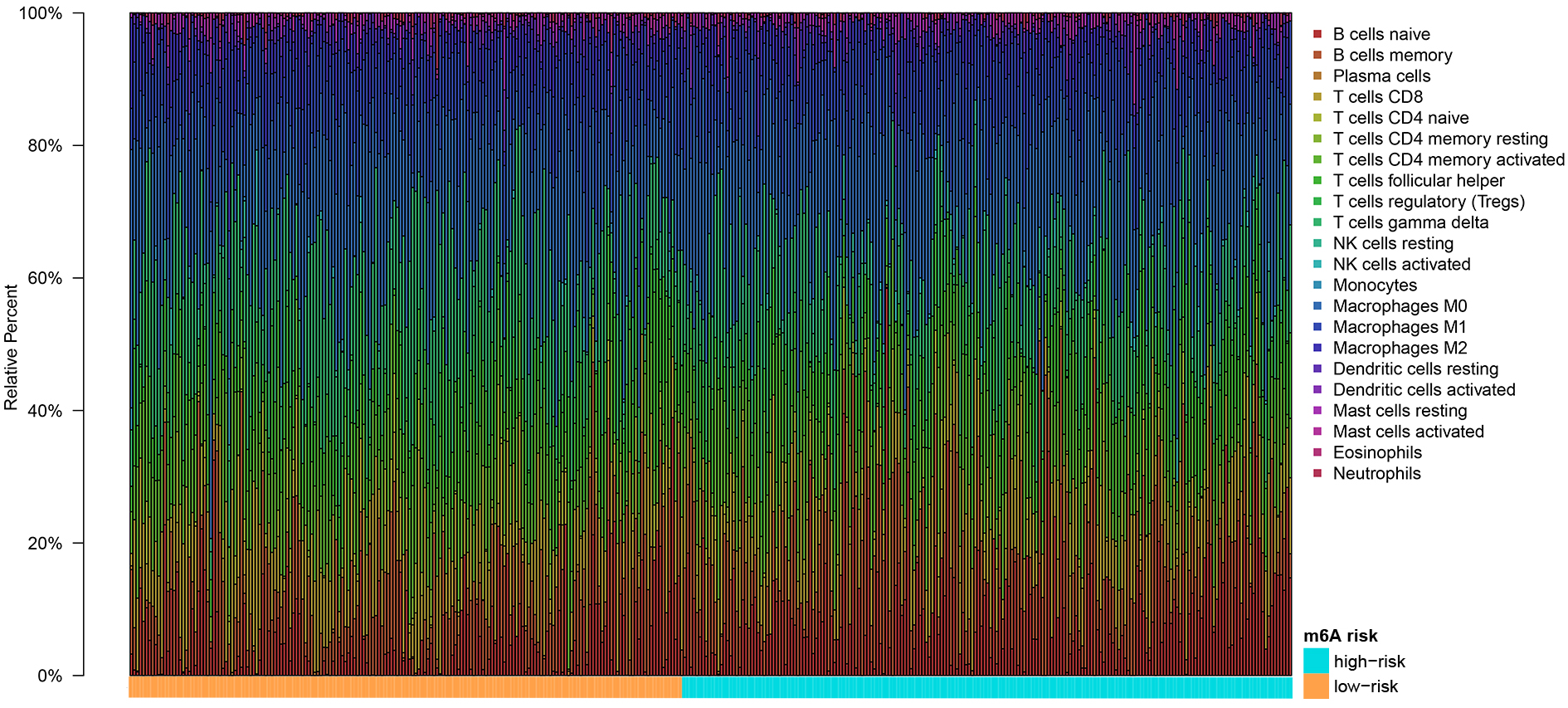

Supplement: Supplemental Material [file KBIE_A_1972644_SM0980.zip › supplementary/Figure S2.jpg]

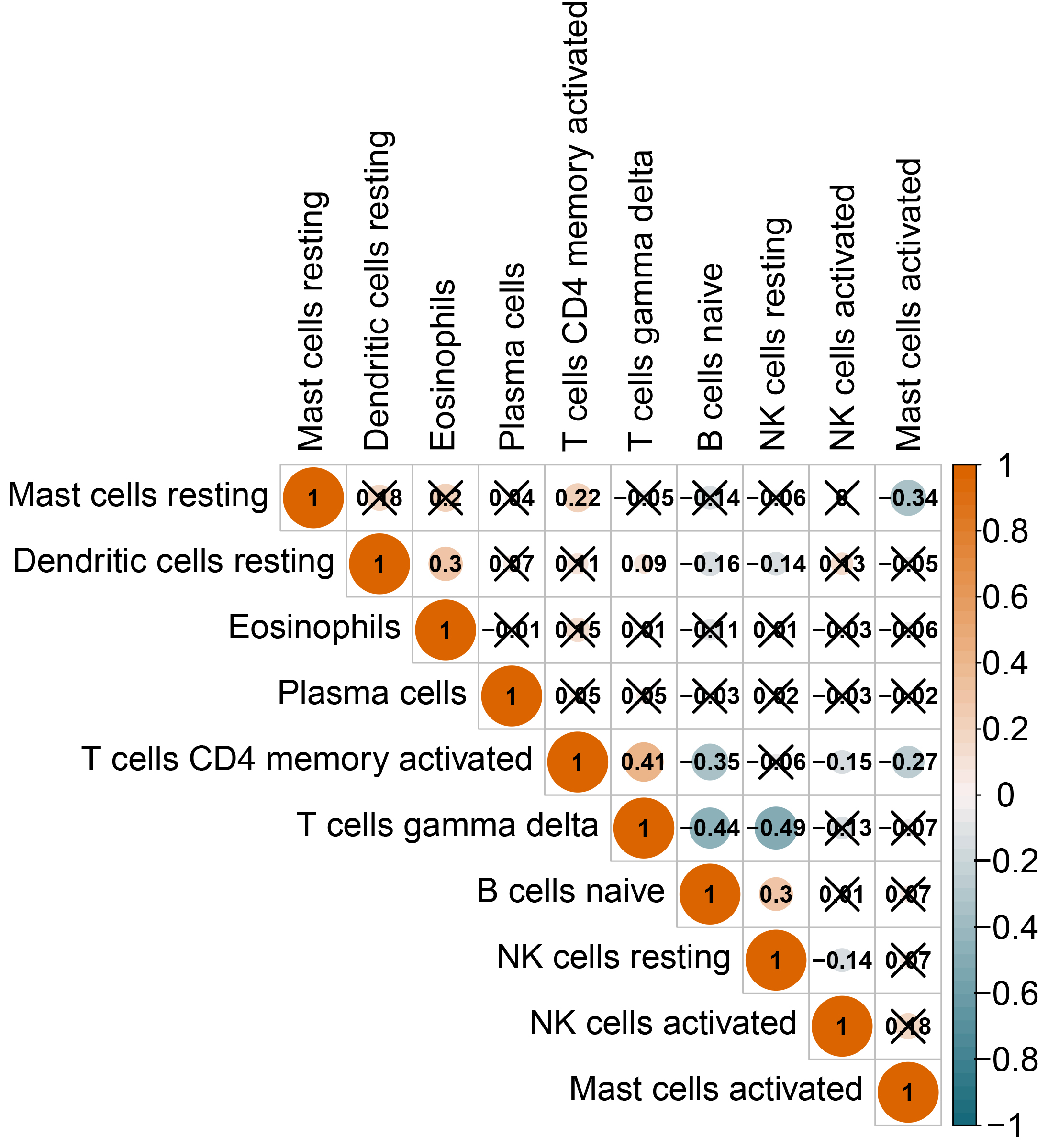

Supplement: Supplemental Material [file KBIE_A_1972644_SM0980.zip › supplementary/Figure S3.jpg]
